# Supplementary figures and images for: Ligand bias underlies differential signaling of multiple FGFs via FGFR1
Source: eLife. 2024 Apr 3;12:RP88144. doi: 10.7554/eLife.88144 (PMC10990489; doi:10.7554/eLife.88144)

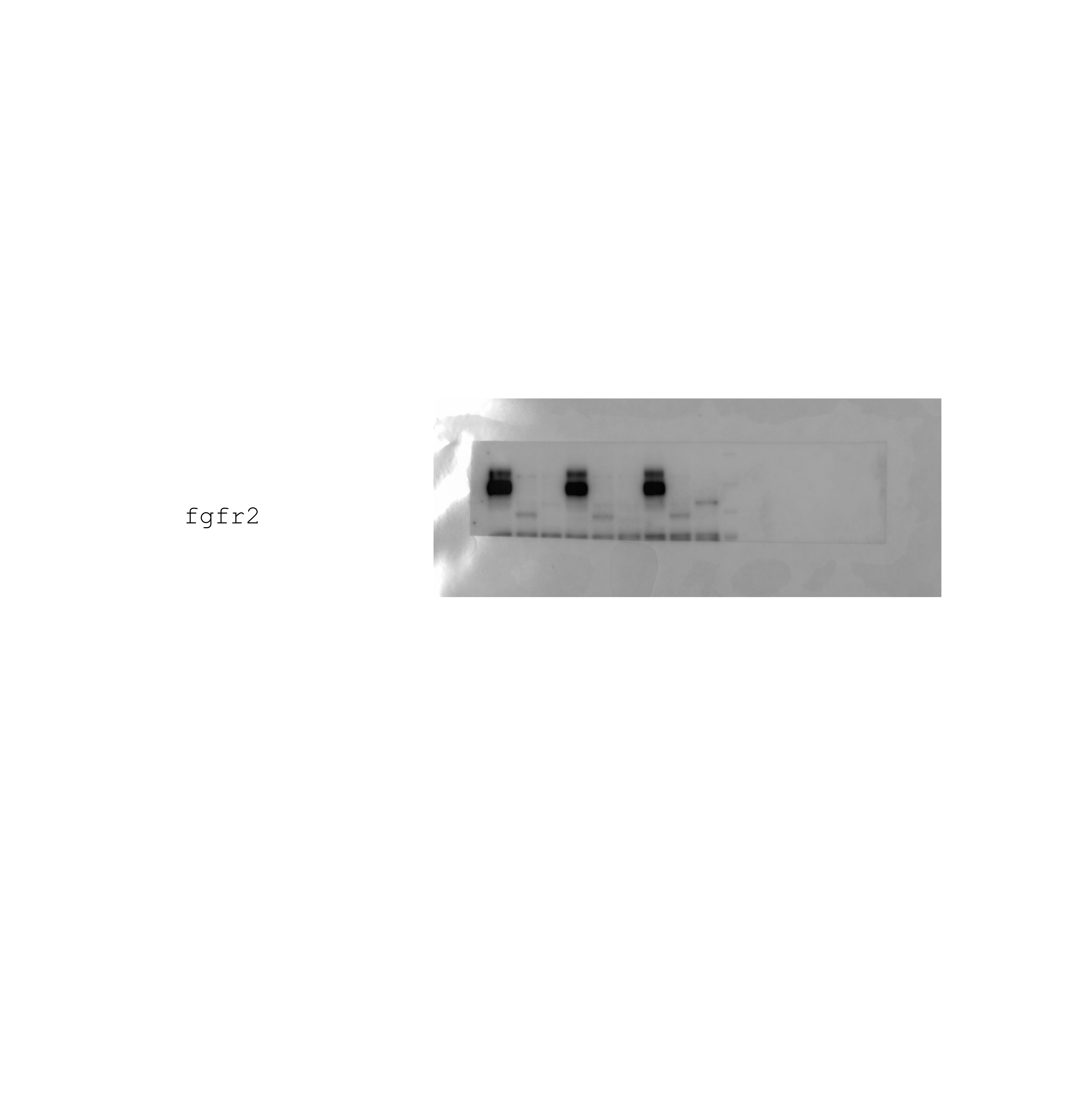

Supplement: Figure 1—source data 1. [file elife-88144-fig1-data1.zip › FGFR2.TIF]

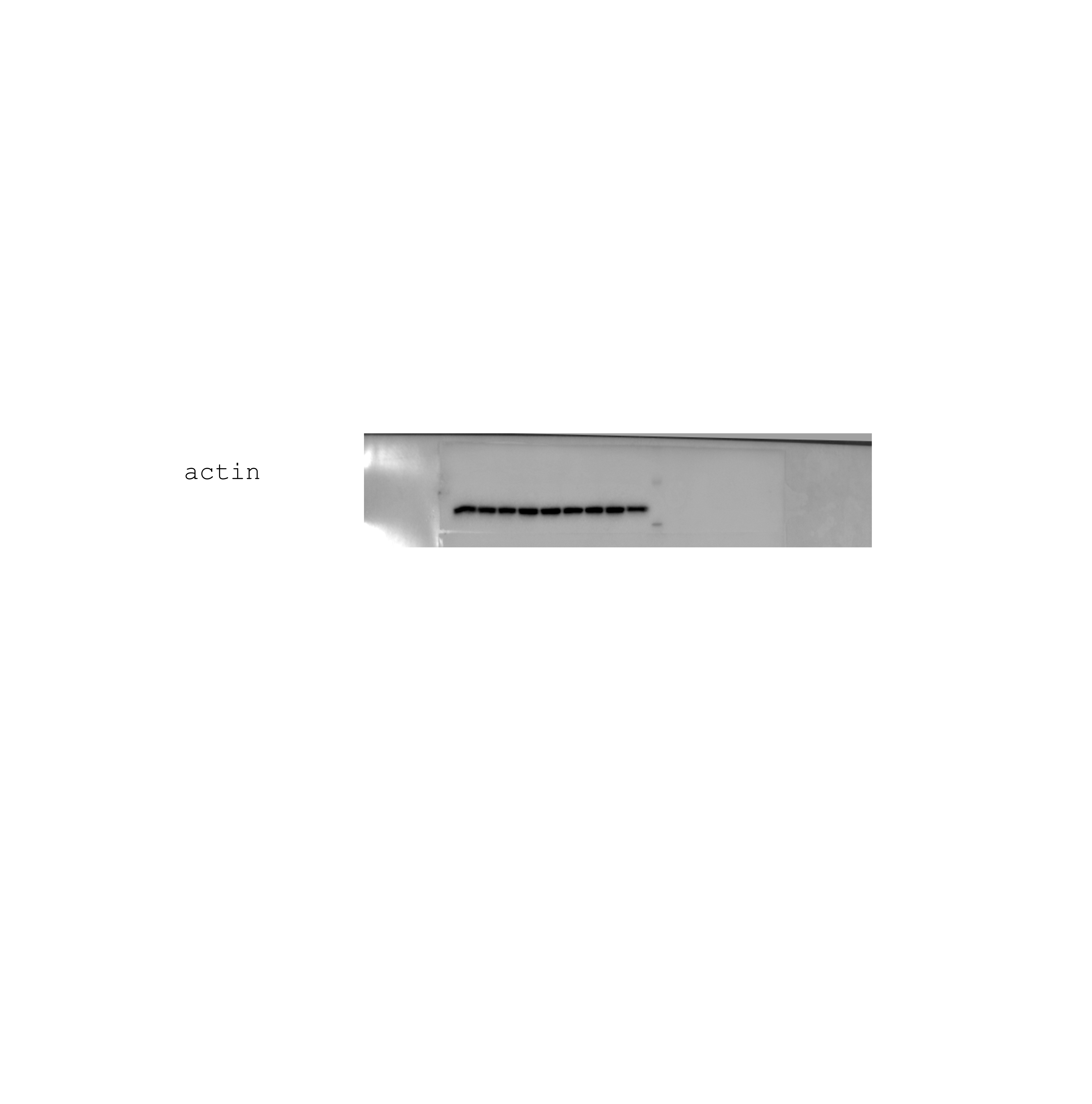

Supplement: Figure 1—source data 1. [file elife-88144-fig1-data1.zip › actin.TIF]

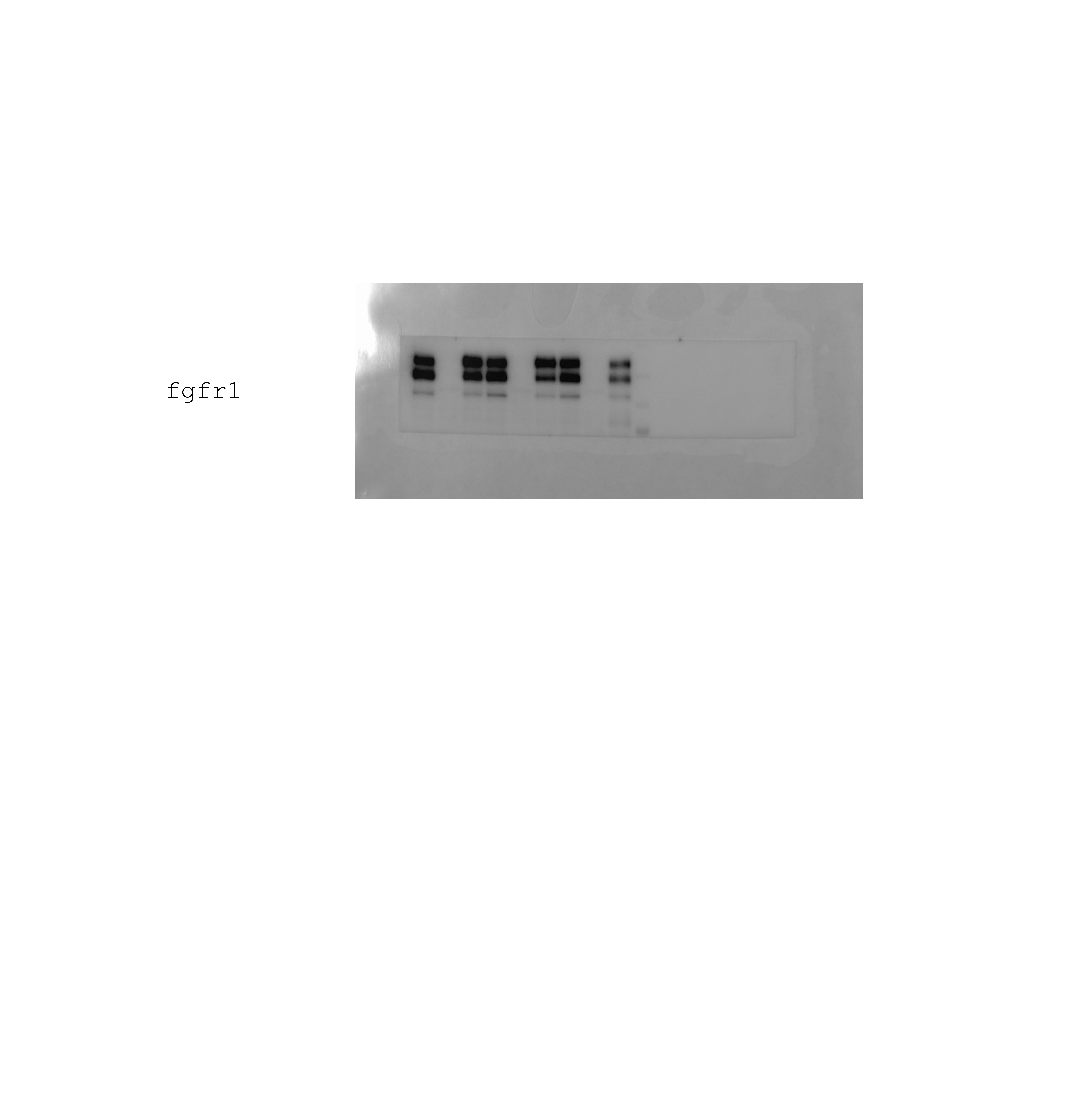

Supplement: Figure 1—source data 1. [file elife-88144-fig1-data1.zip › FGFR1.TIF]

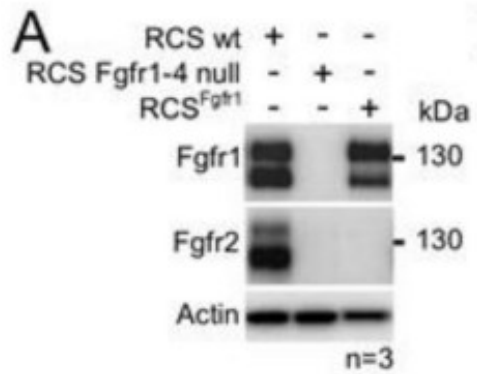

| RCS wt               | + | - | - | + | - | - | + | - | - |
|----------------------|---|---|---|---|---|---|---|---|---|
| RCS Fgfr1-4 null     | - | + | - | - | + | - | - | + | - |
| RCS <sup>Fgfr1</sup> | - | - | + | - | - | + | - | - | + |

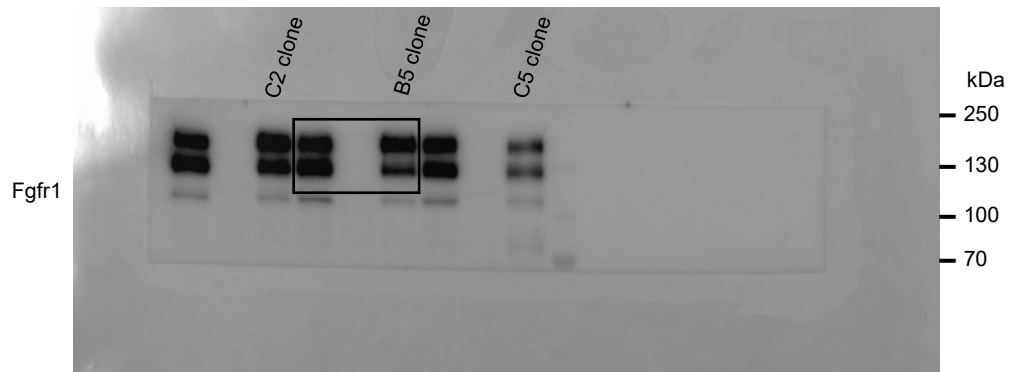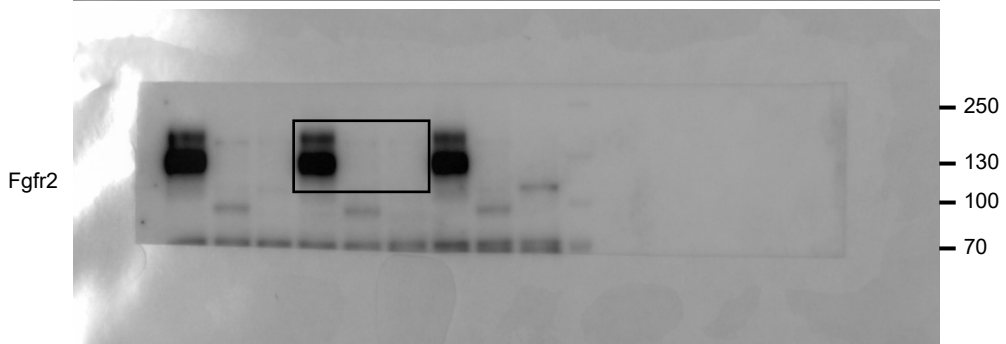

| RCS wt               | + | - | - | + | - | - | + | - | - |
|----------------------|---|---|---|---|---|---|---|---|---|
| RCS Fgfr1-4 null     | - | + | - | - | + | - | - | + | - |
| RCS <sup>Fgfr1</sup> | - | - | + | - | - | + | - | - | + |

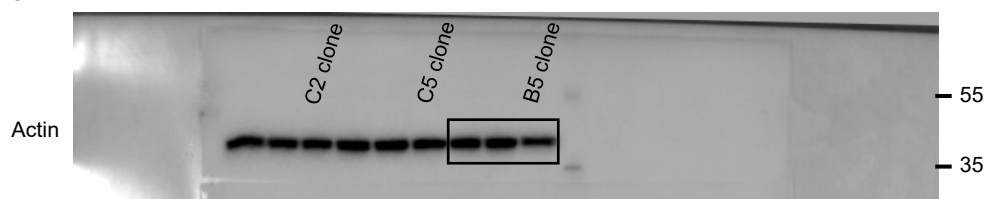

Supplement: Figure 1—source data 2. [file elife-88144-fig1-data2.pdf]

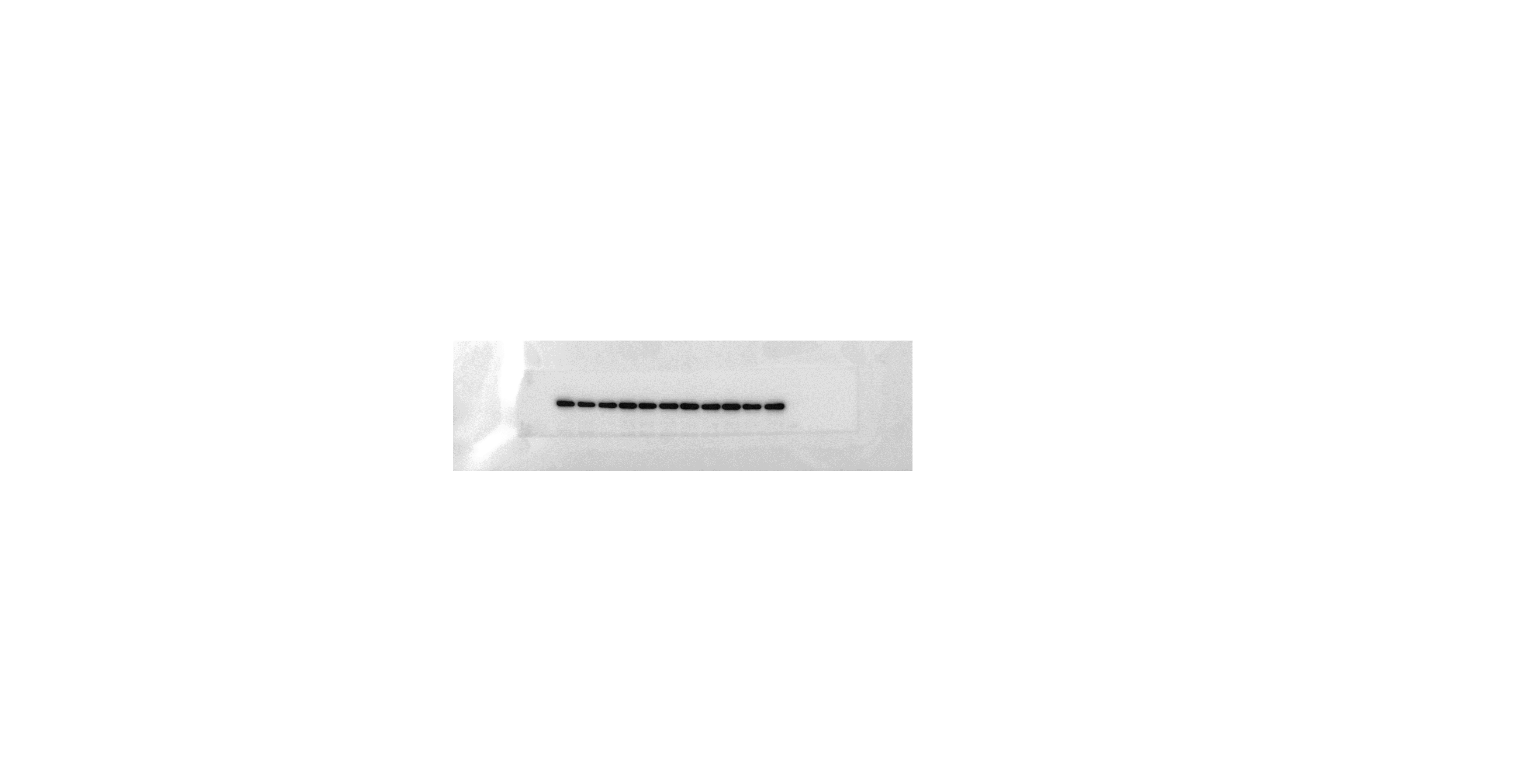

Supplement: Figure 1—source data 3. [file elife-88144-fig1-data3.zip › vinculin.TIF]

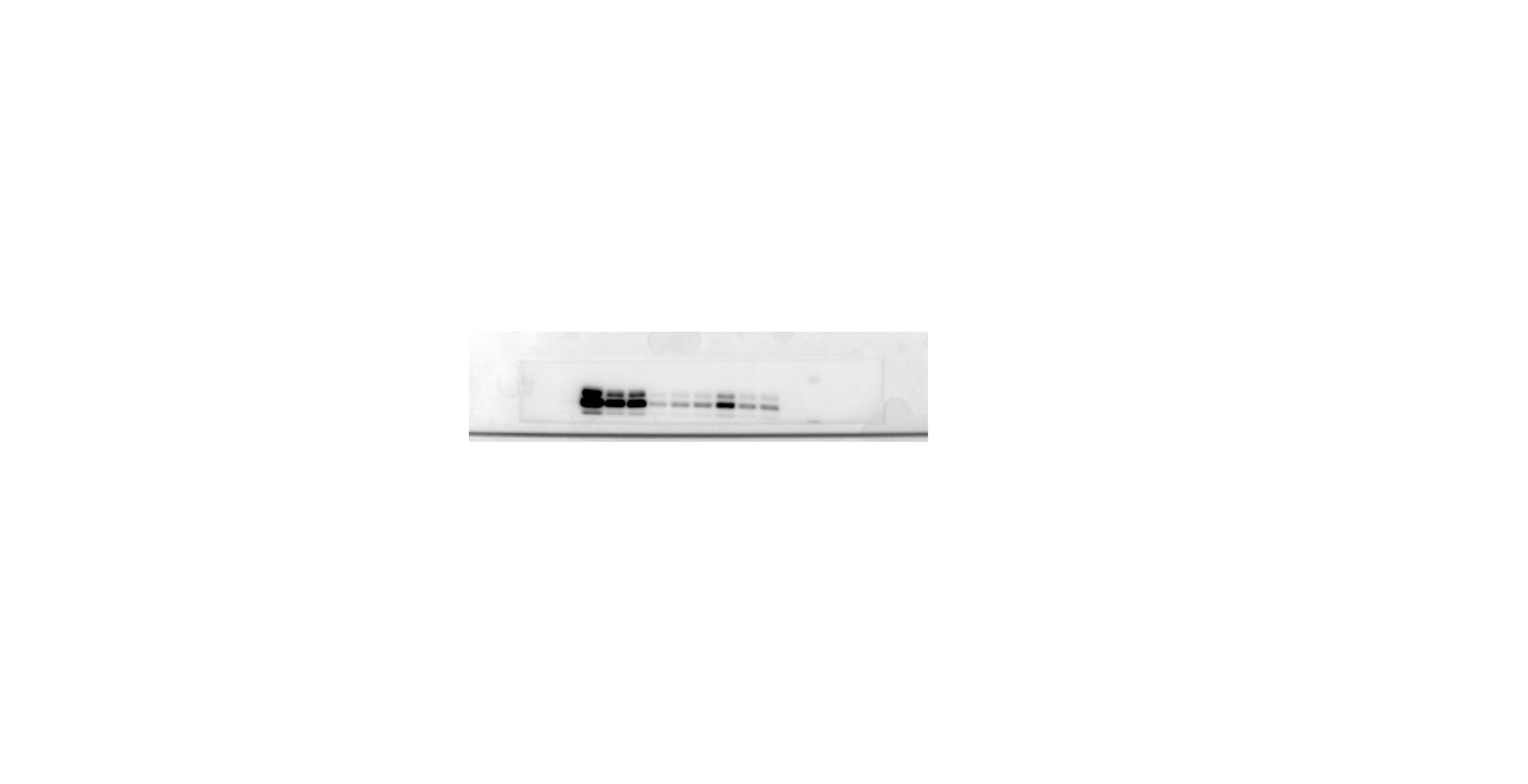

Supplement: Figure 1—source data 3. [file elife-88144-fig1-data3.zip › pERK.TIF]

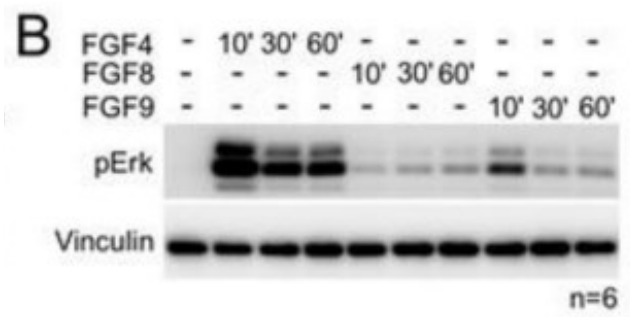

|      |   |     |     |     |     |     |     |     |     |     |
|------|---|-----|-----|-----|-----|-----|-----|-----|-----|-----|
| FGF4 | - | 10' | 30' | 60' | -   | -   | -   | -   | -   | -   |
| FGF8 | - | -   | -   | -   | 10' | 30' | 60' | -   | -   | -   |
| FGF9 | - | -   | -   | -   | -   | -   | -   | 10' | 30' | 60' |

pERK

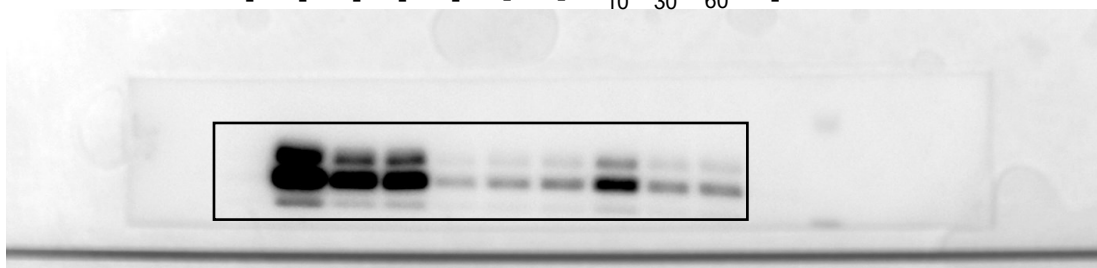

Vinculin

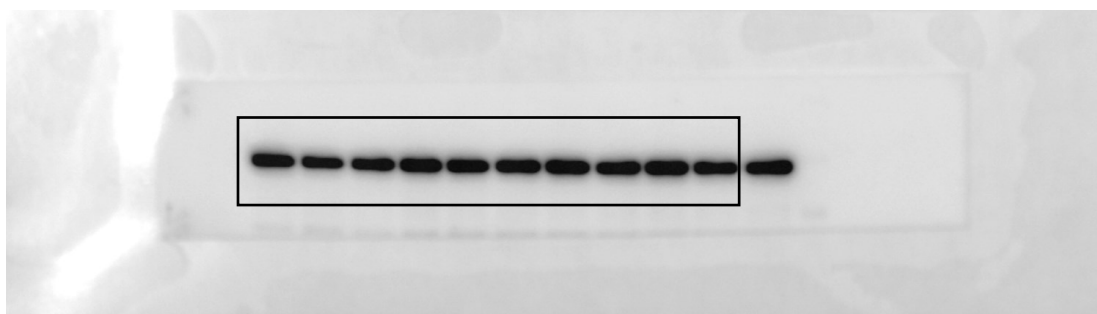

Supplement: Figure 1—source data 4. [file elife-88144-fig1-data4.pdf]

**A**

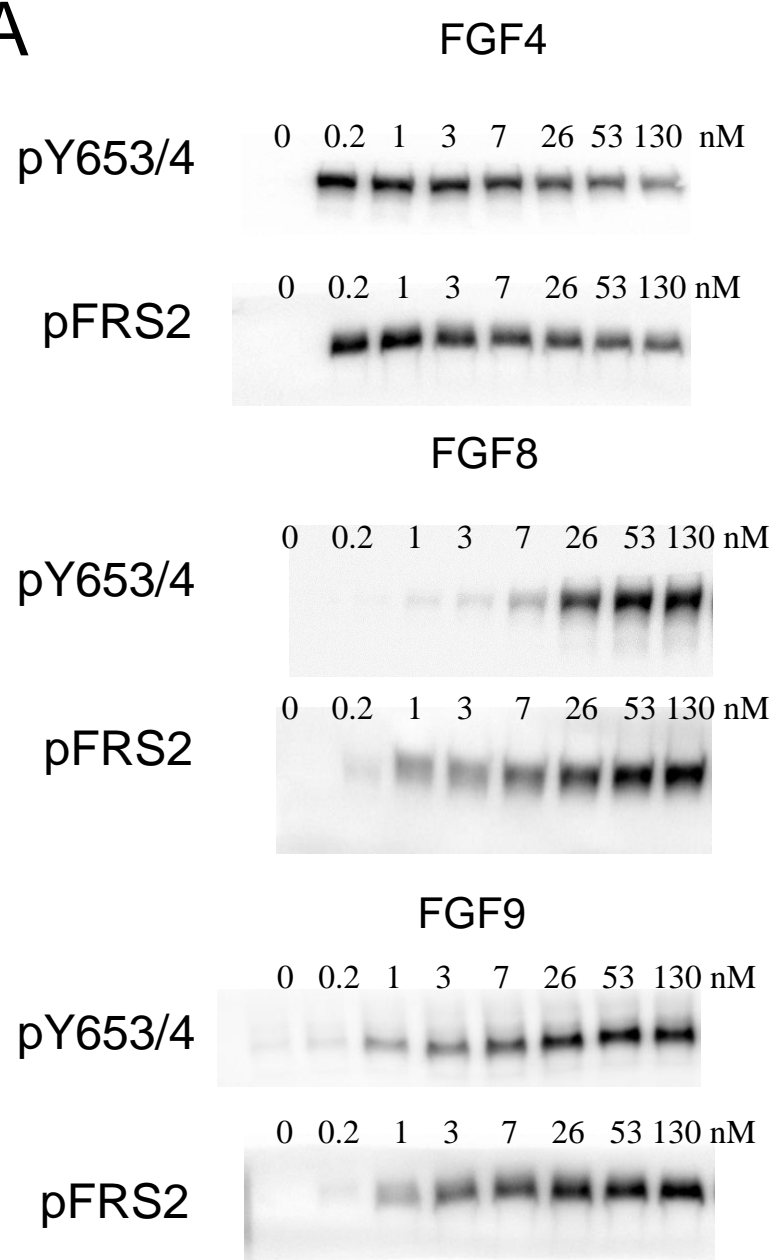

**B**

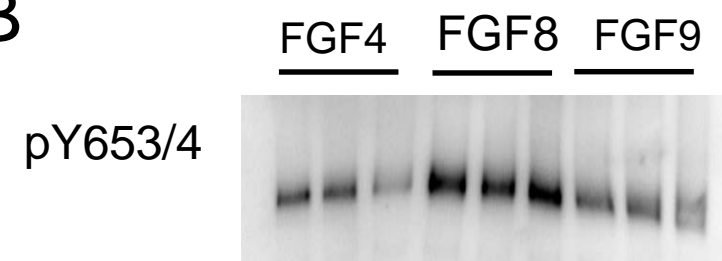

**FGF4**

pY653/4

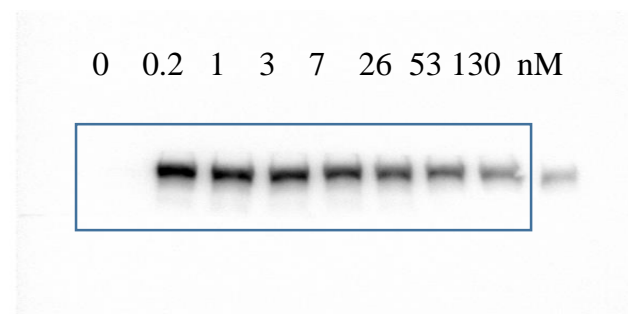

pFRS2

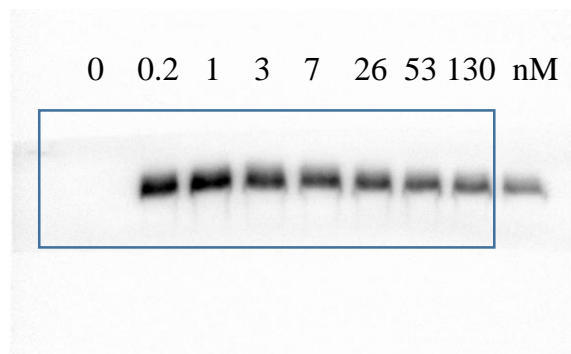

**FGF8**

pY653/4

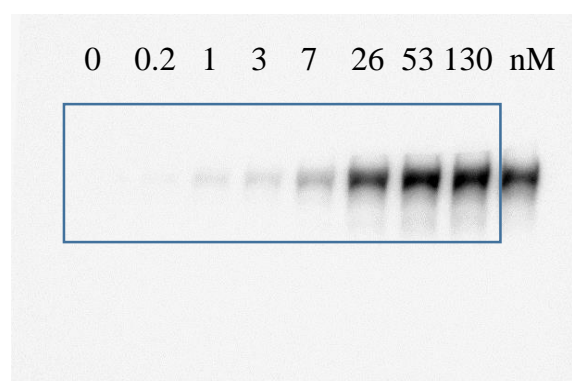

pFRS2

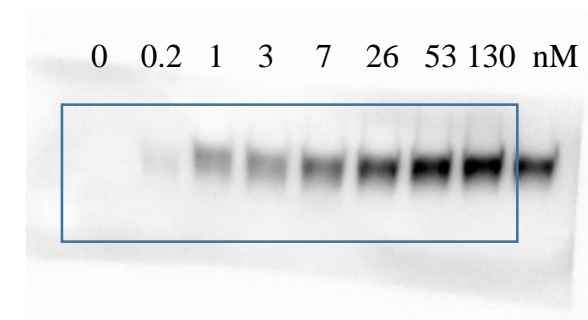

**FGF9**

pY653/4

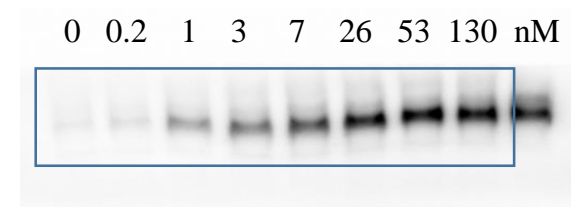

pFRS2

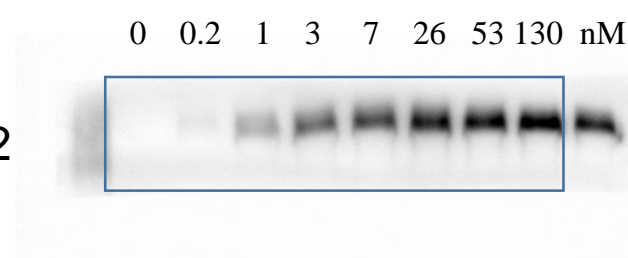

**FGF4** **FGF8** **FGF9**

pY653/4

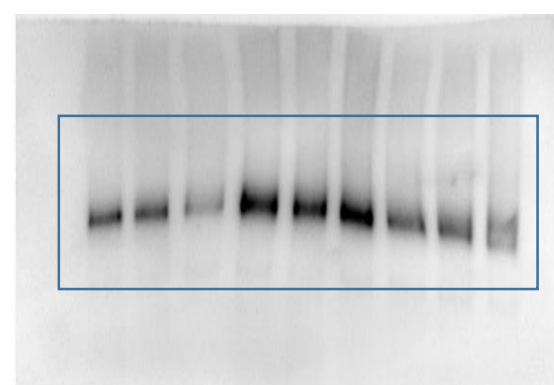

Supplement: Figure 3—source data 1. [file elife-88144-fig3-data1.pdf]

**A**

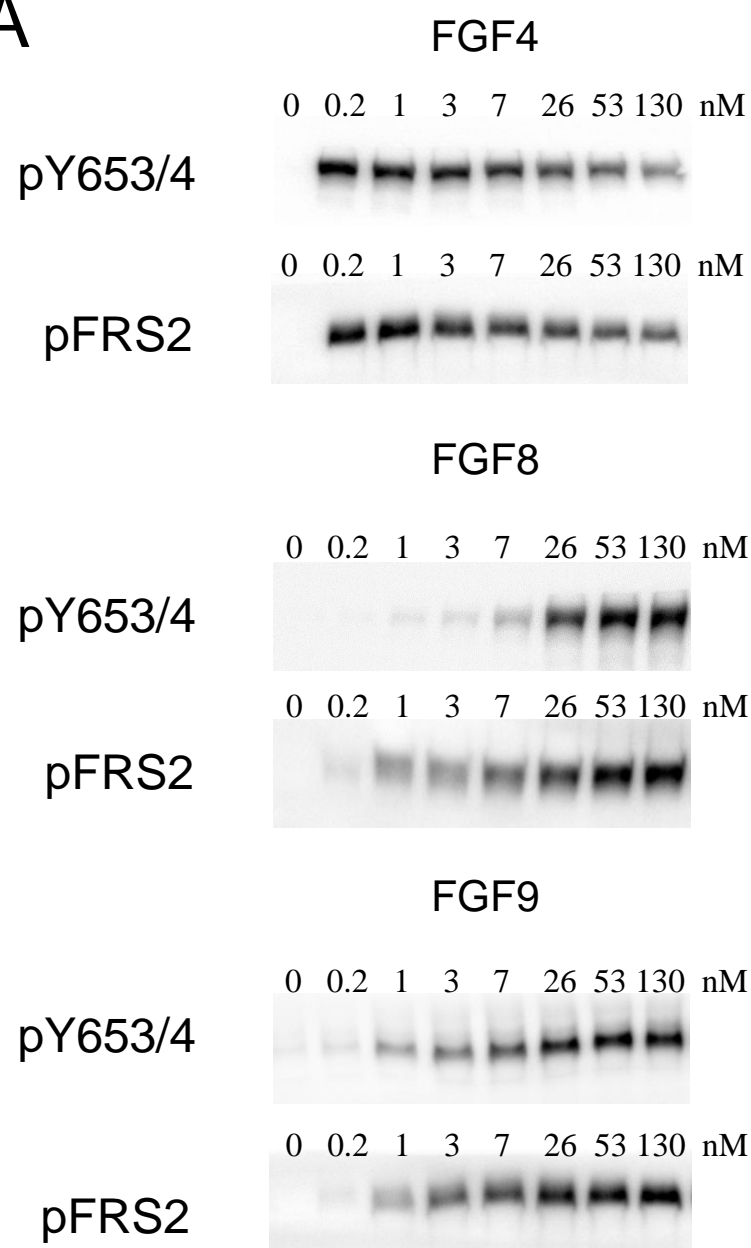

**B**

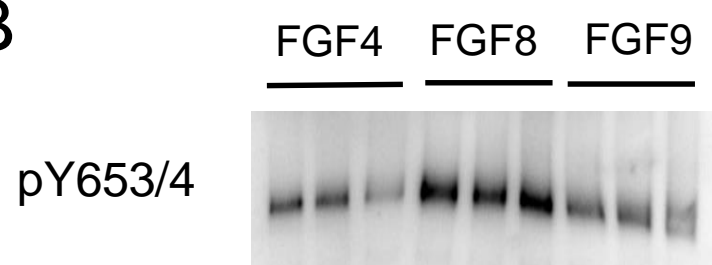

**C**

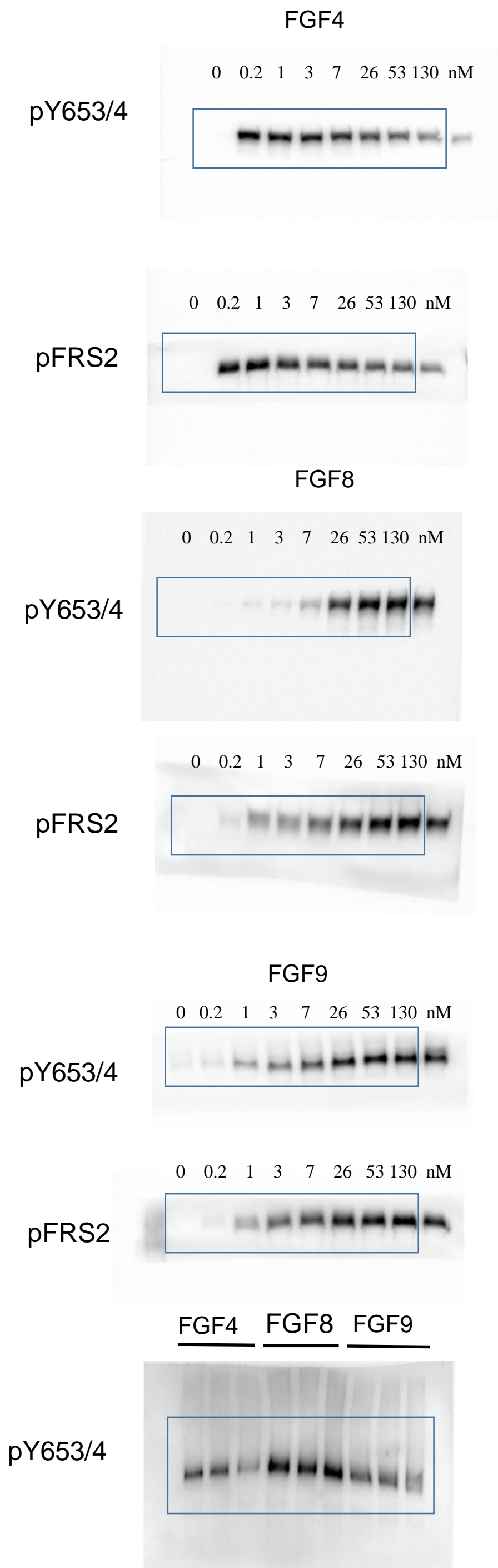

Supplement: Figure 3—source data 2. [file elife-88144-fig3-data2.pdf]

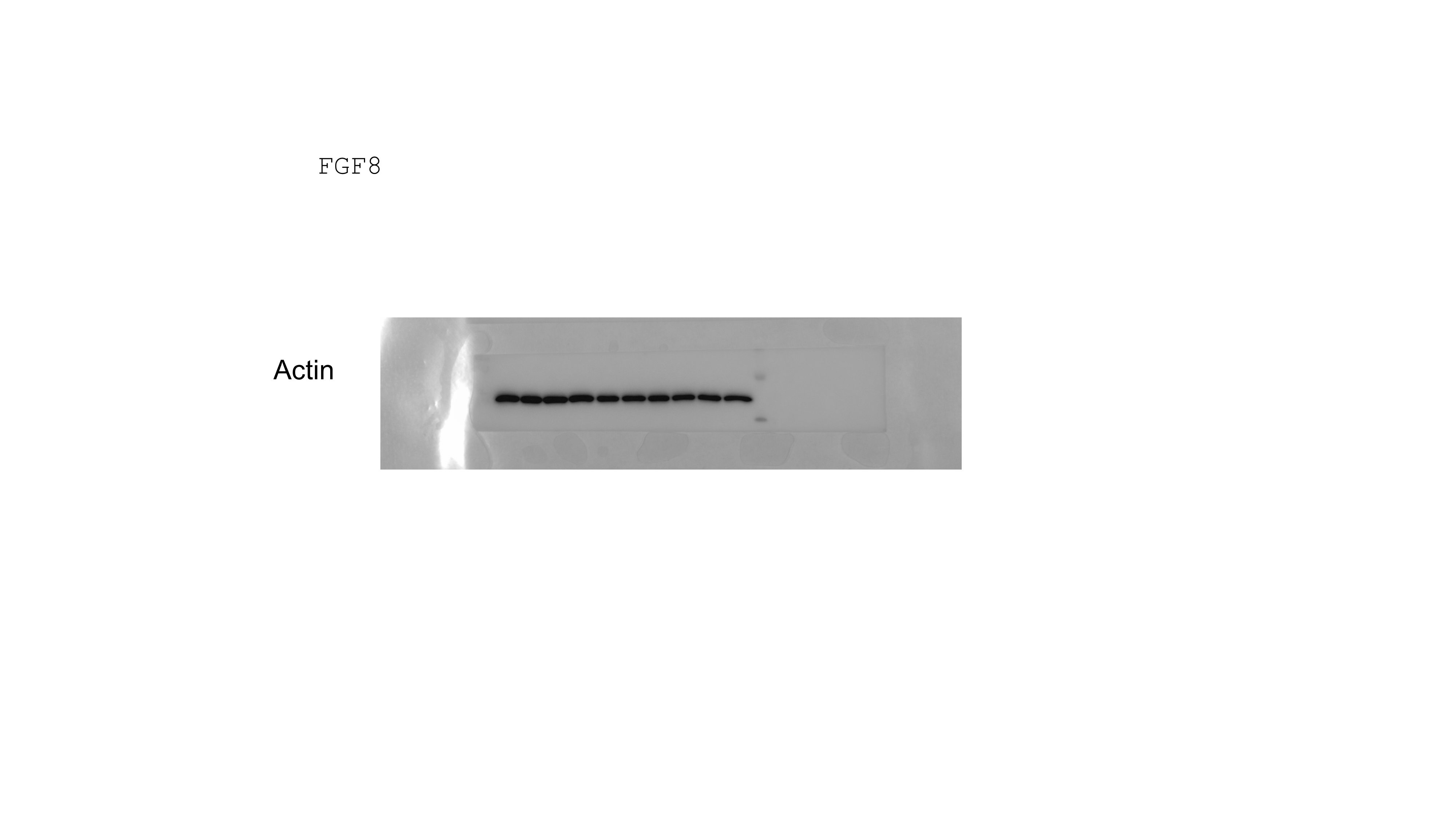

Supplement: Figure 5—source data 2. [file elife-88144-fig5-data2.zip › FGF8actin.TIF]

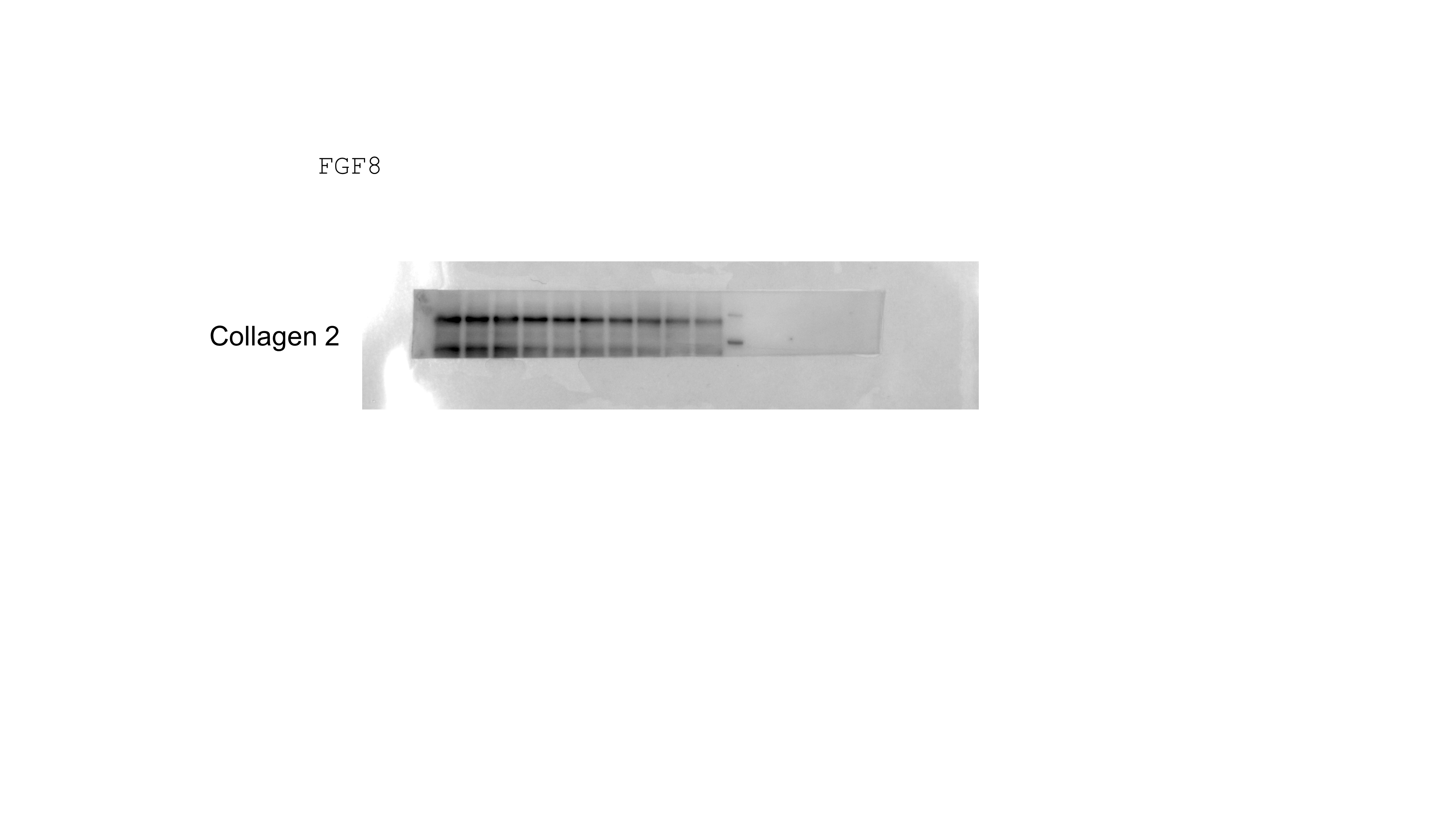

Supplement: Figure 5—source data 2. [file elife-88144-fig5-data2.zip › FGF8Collagen.TIF]

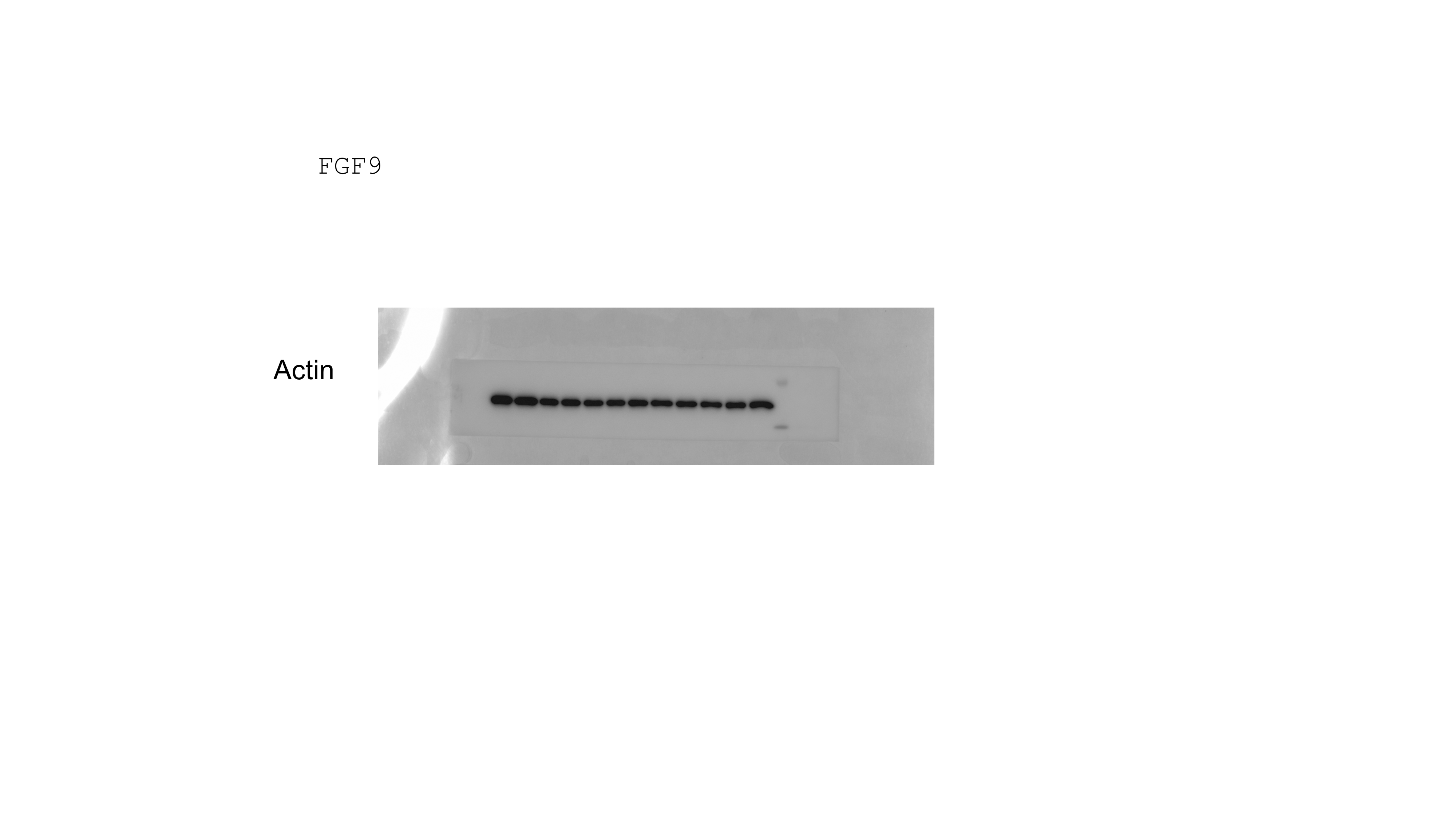

Supplement: Figure 5—source data 2. [file elife-88144-fig5-data2.zip › FGF9Actin.TIF]

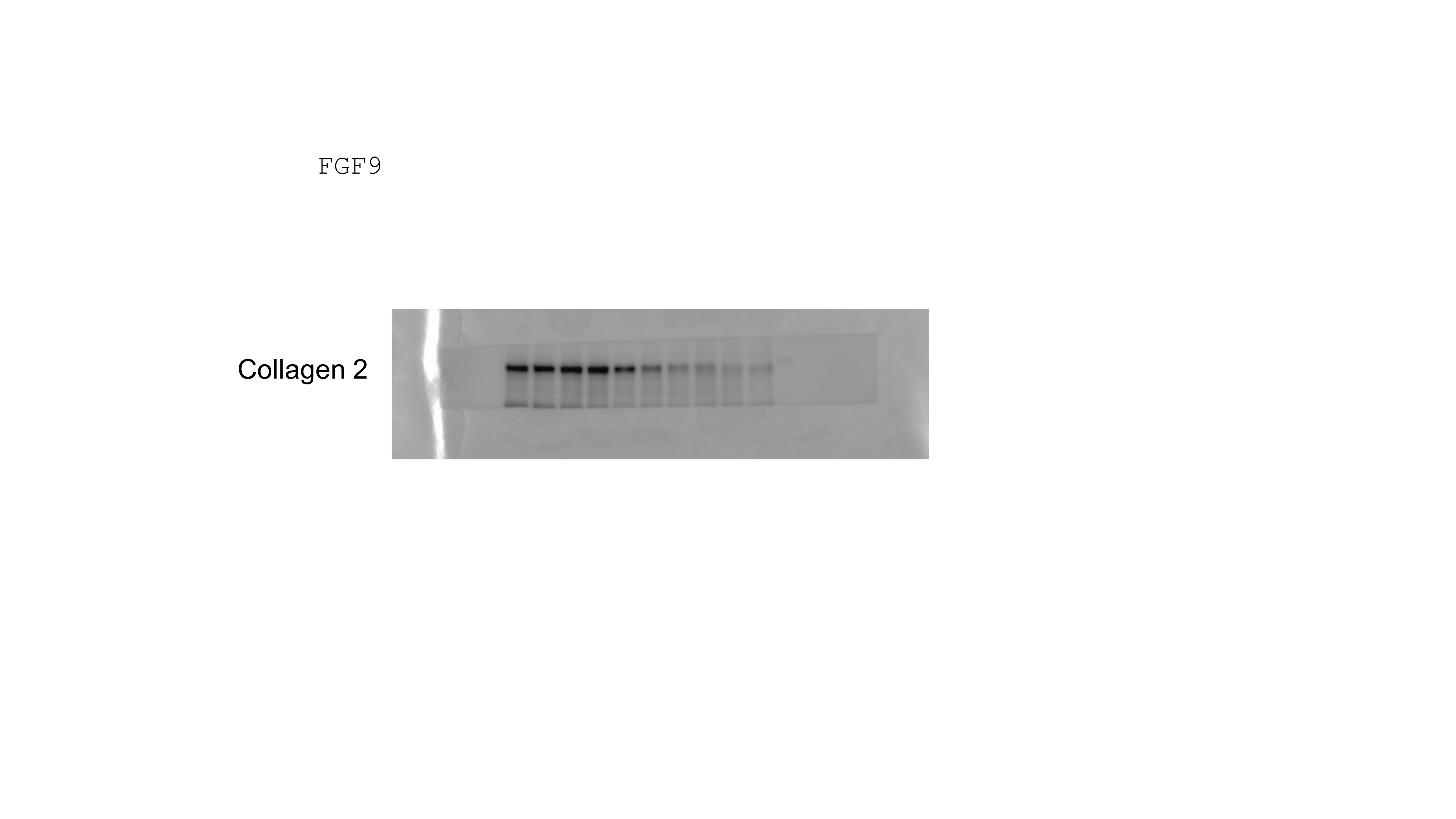

Supplement: Figure 5—source data 2. [file elife-88144-fig5-data2.zip › FGF9collagen.TIF]

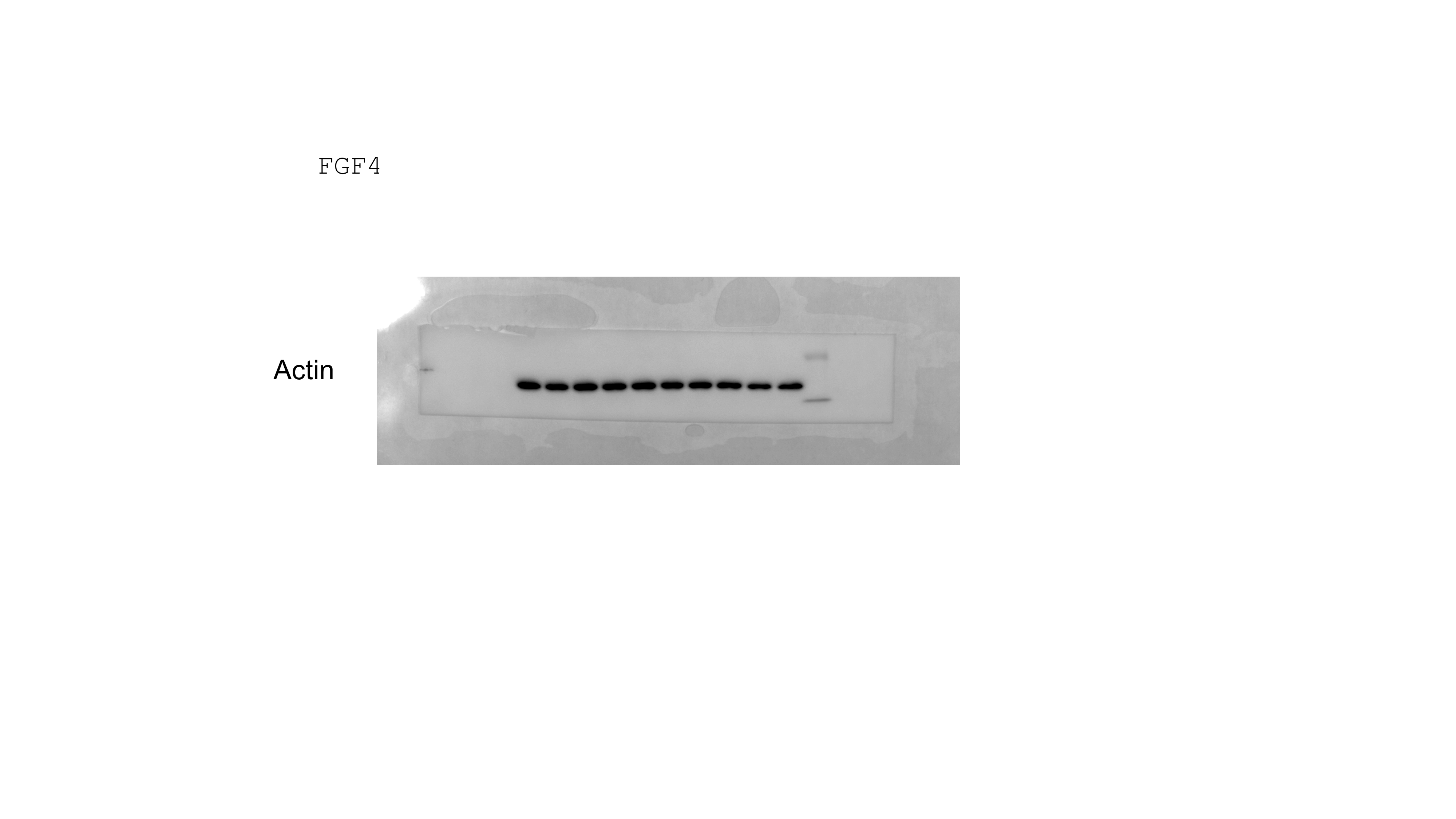

Supplement: Figure 5—source data 2. [file elife-88144-fig5-data2.zip › FGF4Actin.TIF]

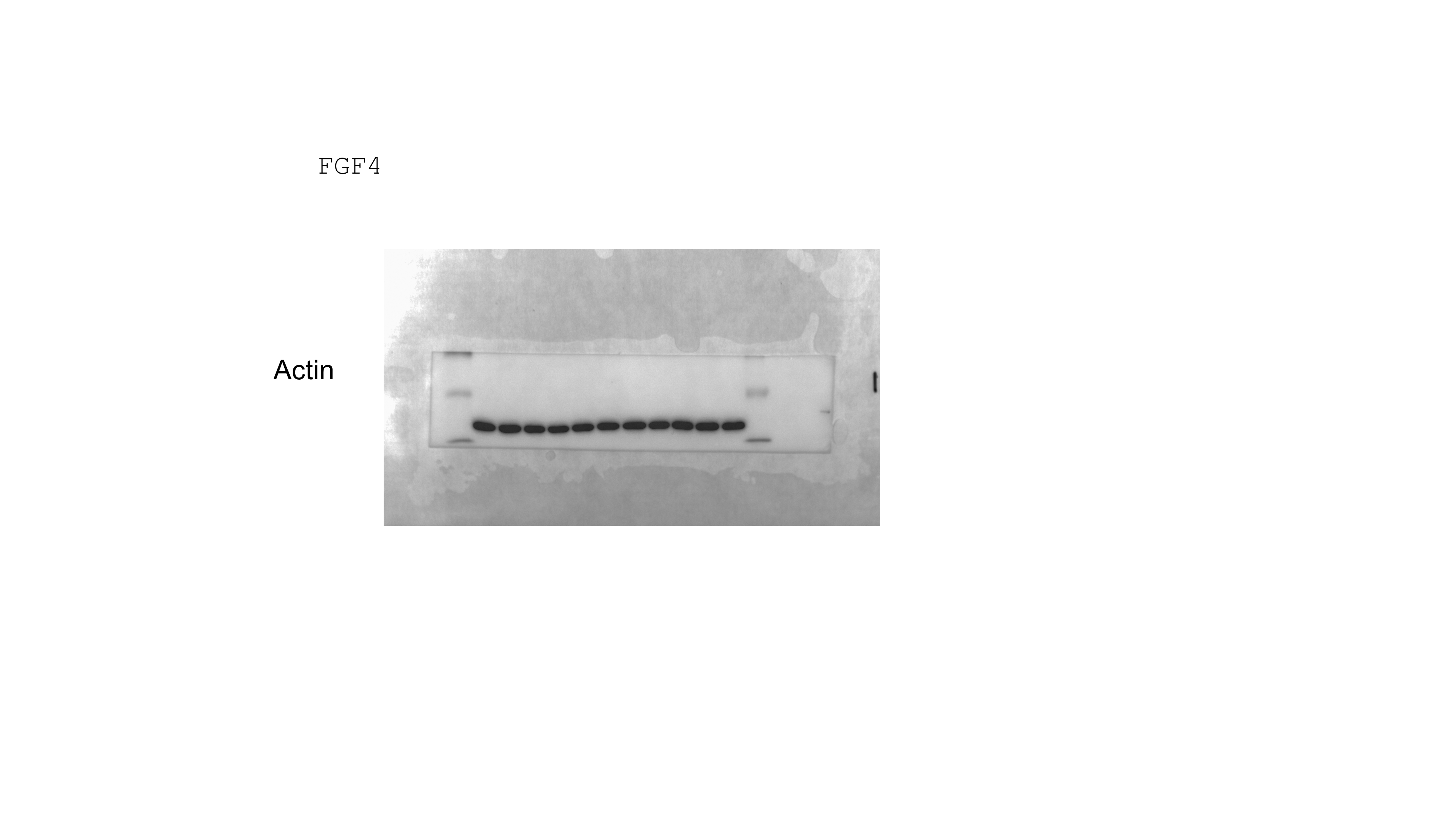

Supplement: Figure 5—source data 2. [file elife-88144-fig5-data2.zip › FGF4Actin2.TIF]

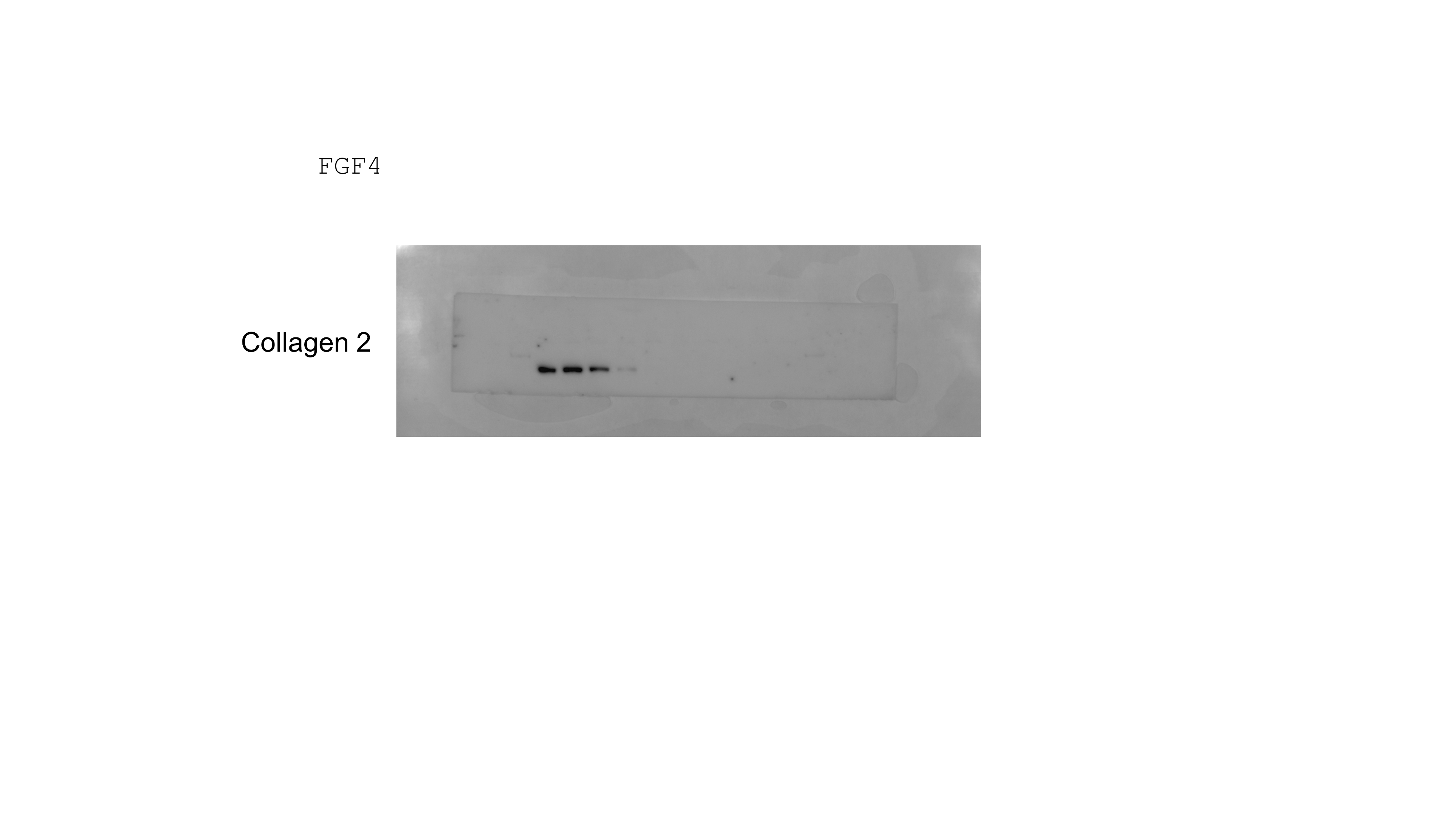

Supplement: Figure 5—source data 2. [file elife-88144-fig5-data2.zip › FGF4Collagen.TIF]

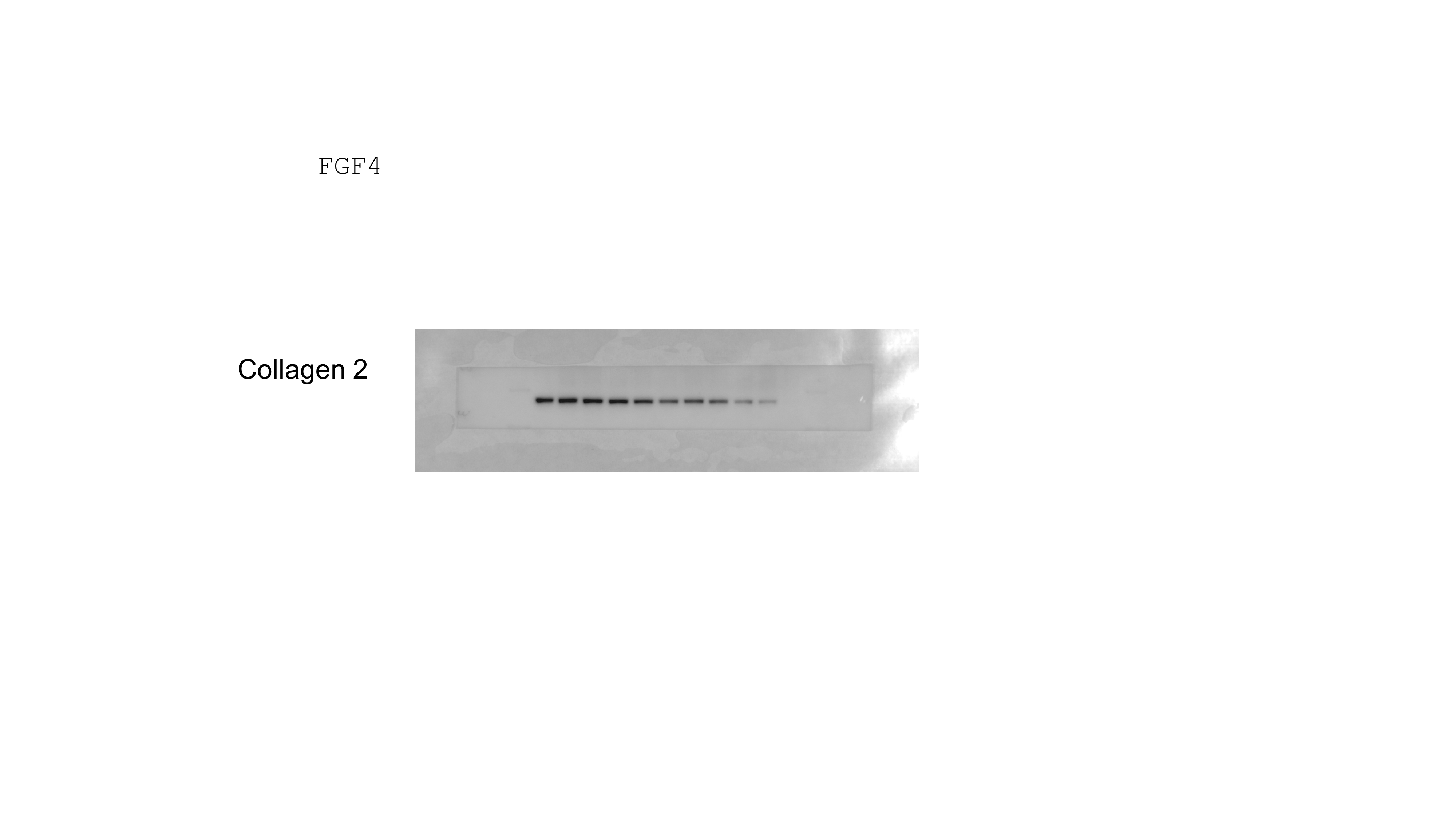

Supplement: Figure 5—source data 2. [file elife-88144-fig5-data2.zip › FGF4Collagen2.TIF]

D

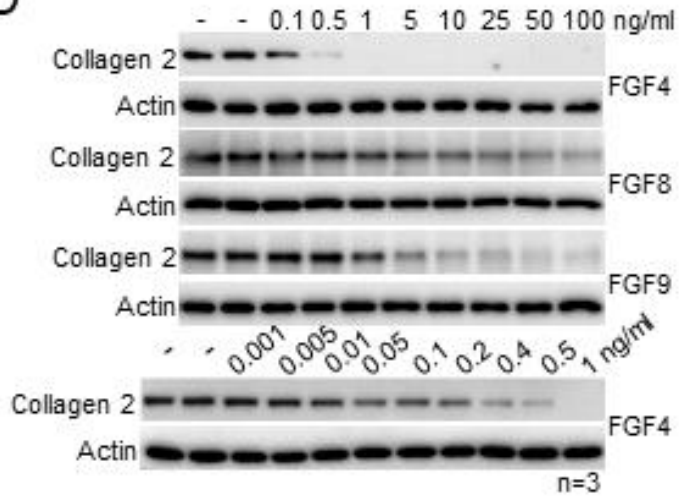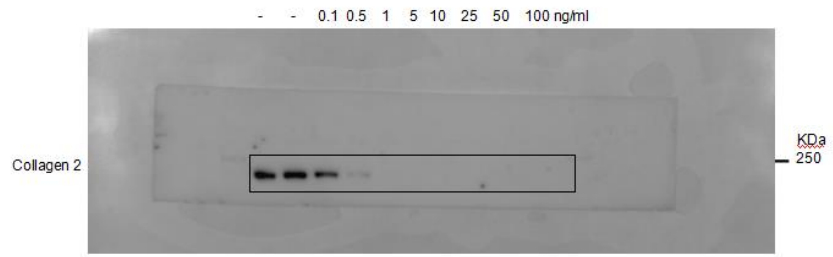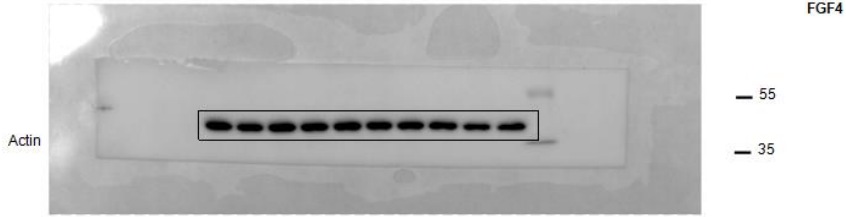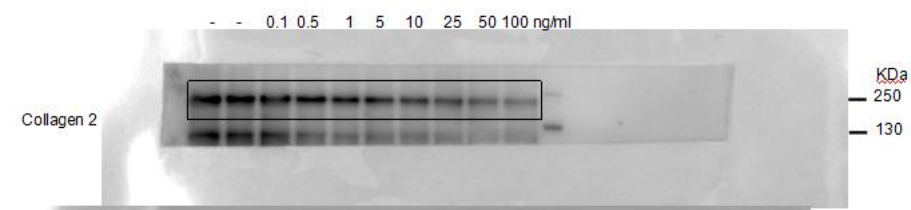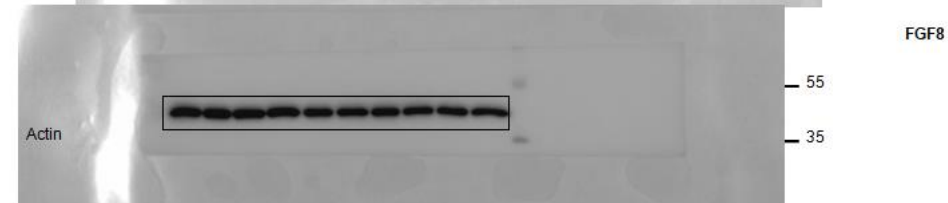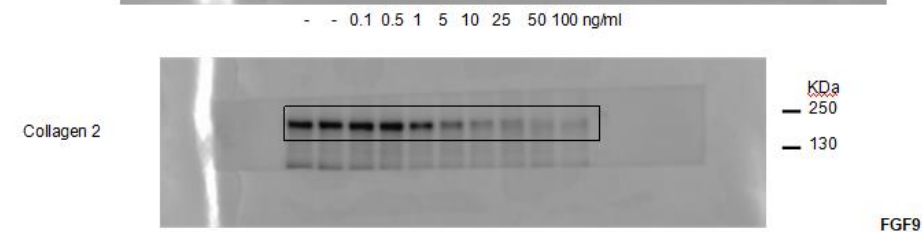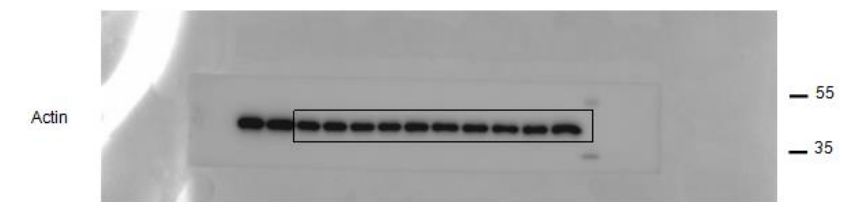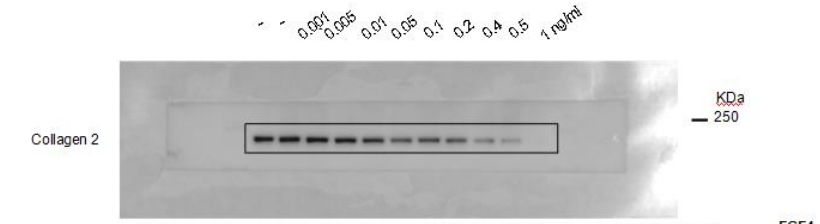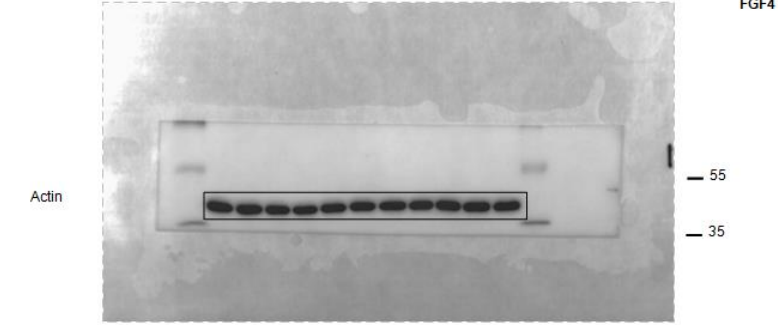

Supplement: Figure 5—source data 3. [file elife-88144-fig5-data3.pdf]

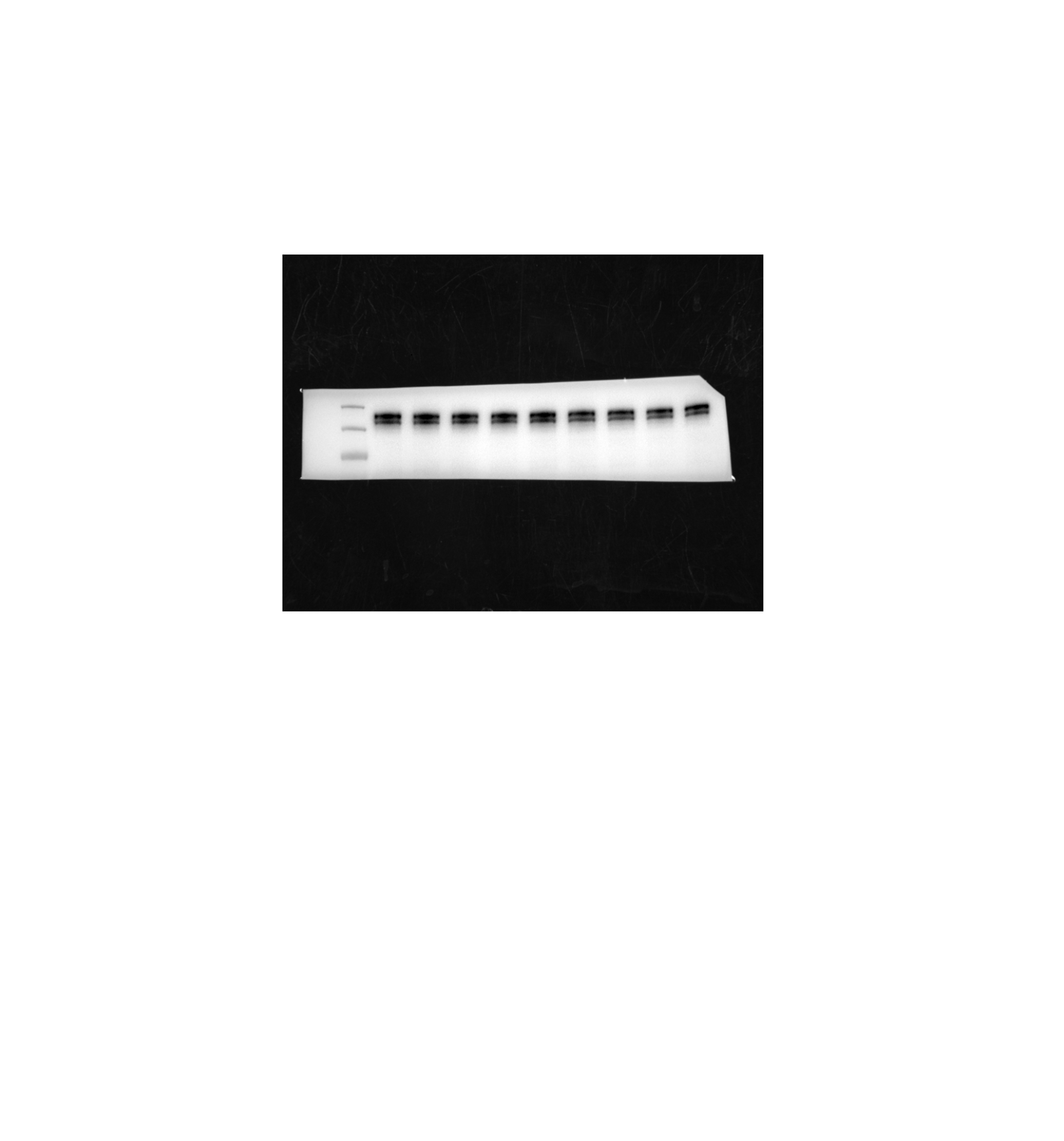

Supplement: Figure 5—figure supplement 1—source data 1. [file elife-88144-fig5-figsupp1-data1.zip › FGFR1.TIF]

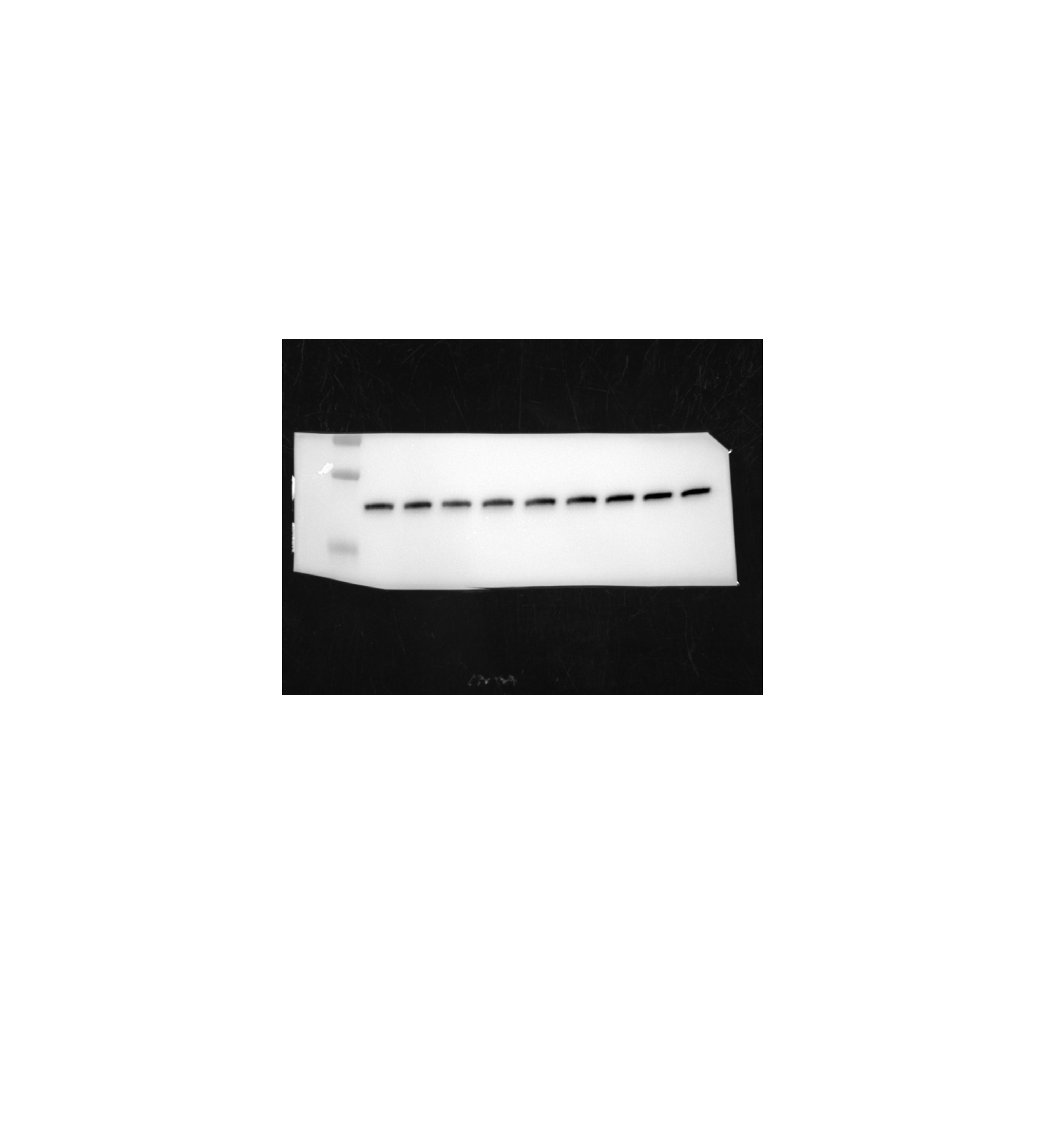

Supplement: Figure 5—figure supplement 1—source data 1. [file elife-88144-fig5-figsupp1-data1.zip › ACTIN.TIF]

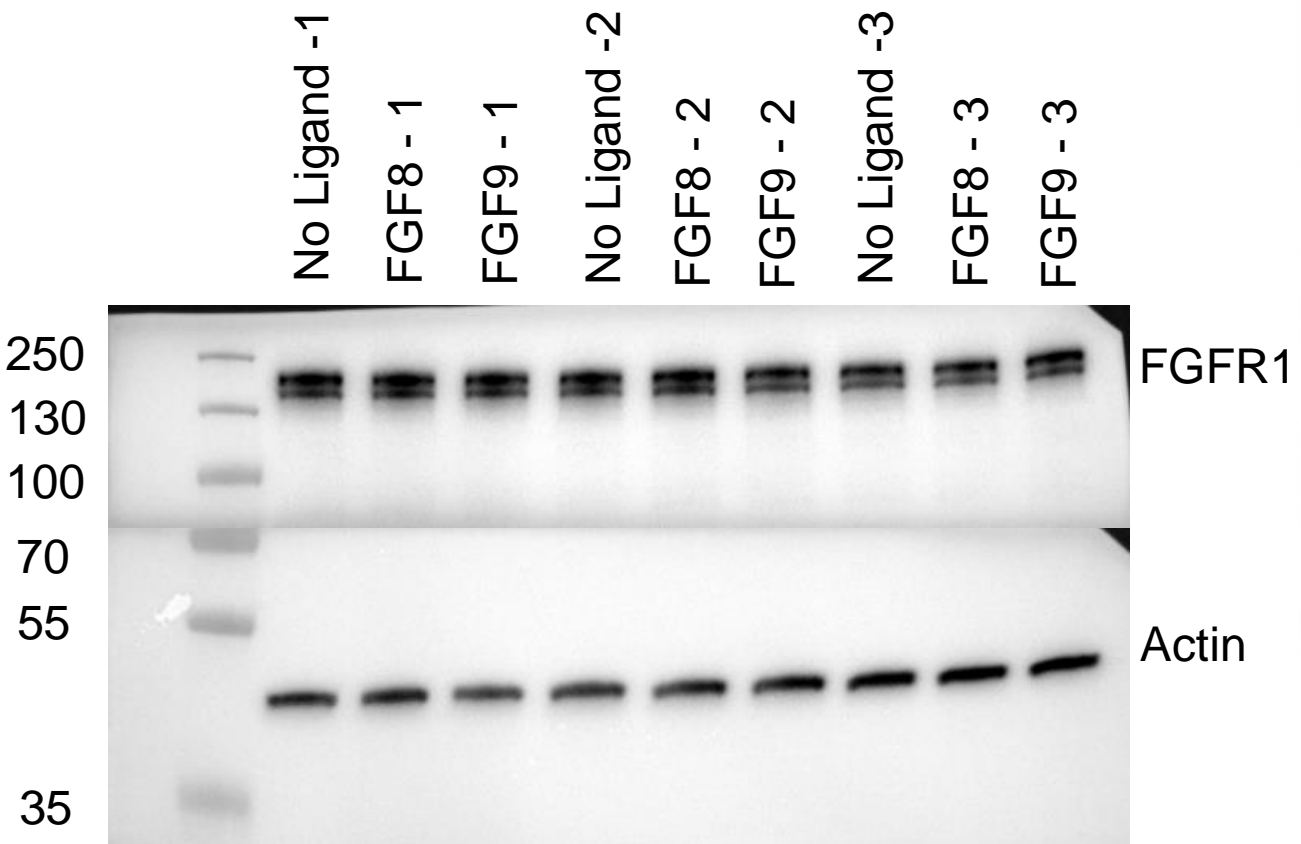

Actin

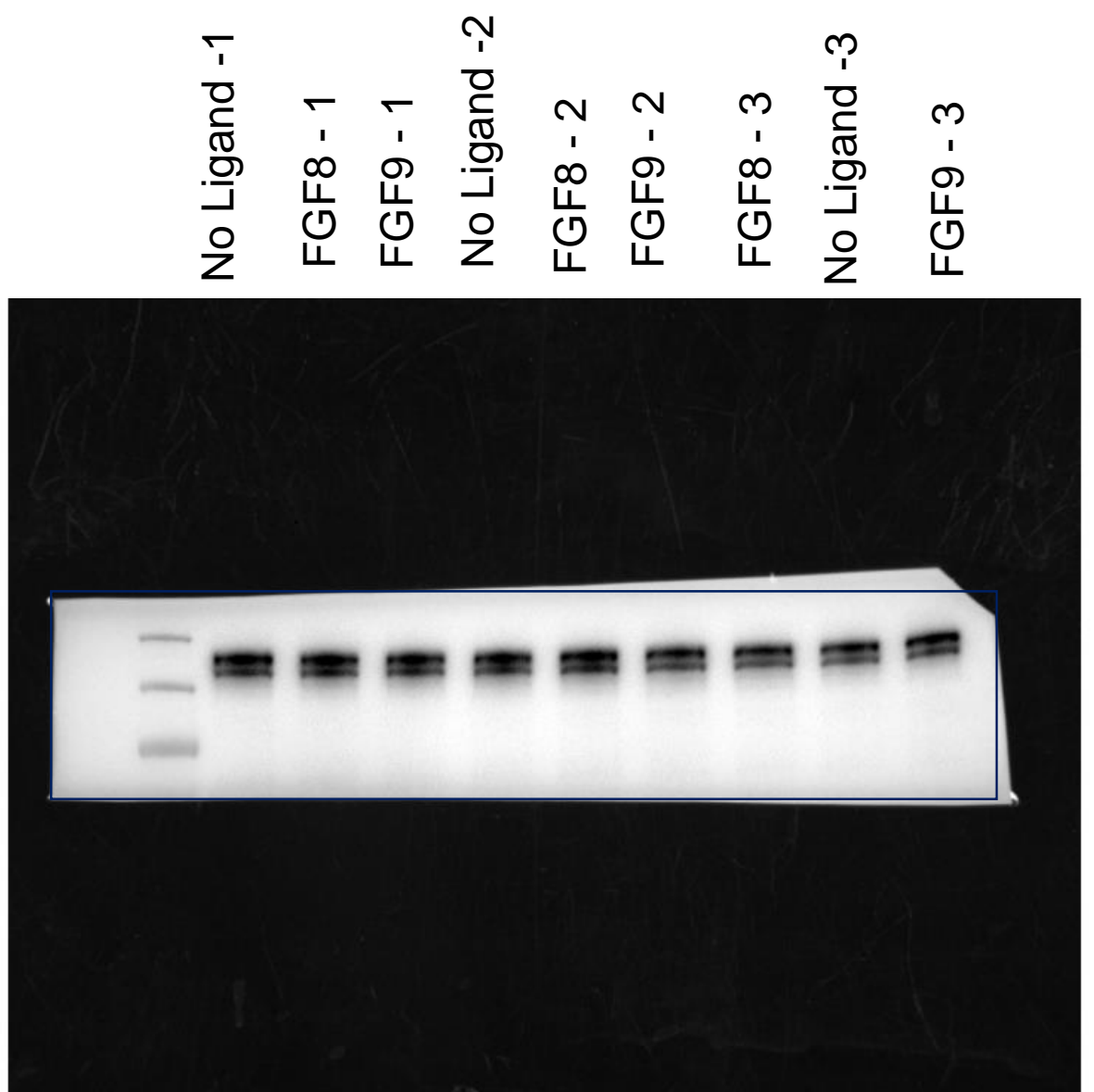

Supplement: Figure 5—figure supplement 1—source data 2. [file elife-88144-fig5-figsupp1-data2.pdf]
