# Supplementary material for: Ligand bias underlies differential signaling of multiple FGFs via FGFR1
Source: eLife. 2024 Apr 3;12:RP88144. doi: 10.7554/eLife.88144 (PMC10990489; doi:10.7554/eLife.88144)
Supplement: Supplementary file 1. [file elife-88144-supp1.docx]

Supplementary table 1: Best-fit Gaussian Parameters for the different log(brightness) distributions

| Gaussian Fit Parameters | | |
| --- | --- | --- |
|  | mean | stdev |
| FGFR1 + 130 nM FGF9 | 0.41 ± 0.01 | 0.30 |
| FGFR1 + 130 nM FGF4 | 0.48 ± 0.01 | 0.31 |
| FGFR1 + 3 nM FGF4 | 0.30 ± 0.01 | 0.22 |
| FGFR1 + 130 nM FGF8 | 0.40 ± 0.01 | 0.25 |
| LAT | 0.22 ± 0.01 | 0.32 |
| TrkA + 130nM NT3 | 0.39 ± 0.01 | 0.29 |
| FGFR1 no ligand | 0.25 ± 0.01 | 0.31 |
