# Supplementary material for: Ligand bias underlies differential signaling of multiple FGFs via FGFR1
Source: eLife. 2024 Apr 3;12:RP88144. doi: 10.7554/eLife.88144 (PMC10990489; doi:10.7554/eLife.88144)
Supplement: Supplementary file 2. [file elife-88144-supp2.docx]

Supplementary table 2: Calculated Z values from the Gaussian fit parameters in supplementary File 1. Z>2 indicates statistically significant differences (37).

| Z values for Gaussians | | | | | | |
| --- | --- | --- | --- | --- | --- | --- |
|  | FGFR1 +  130nM FGF4 | FGFR1 + 3nM FGF4 | FGFR1 + 130nM FGF8 | LAT | TrkA + 130nM NT3 | FGFR1  no ligand |
| FGFR1 + 130nM FGF9 | 2.664092 | 4.477257 | 0.318121 | 5.901904 | 0.436884 | 4.868914751 |
| FGFR1 + 130nM FGF4 |  | 10.4334 | 3.379447 | 9.628828 | 3.421783 | 7.801433959 |
| FGFR1 + 3nM FGF4 |  |  | 4.768932 | 3.348705 | 3.221729 | 3.221729253 |
| FGFR1 + 130nM FGF8 |  |  |  | 6.089074 | 0.138045 | 4.910927558 |
| LAT |  |  |  |  | 5.825875 | 0.37353812 |
| TrkA+130nM NT3 |  |  |  |  |  | 4.24156545 |
