# Supplementary material for: Ligand bias underlies differential signaling of multiple FGFs via FGFR1
Source: eLife. 2024 Apr 3;12:RP88144. doi: 10.7554/eLife.88144 (PMC10990489; doi:10.7554/eLife.88144)
Supplement: Supplementary file 3. [file elife-88144-supp3.docx]

Supplementary table 3: β’_4_, β’_8_, and β’_9_ values calculated for each response using equation 4

| FGF4 | | | | | |
| --- | --- | --- | --- | --- | --- |
|  | pY653/654 | pY766 | pPLCγ | pFRS2 | Downregulation |
| pY653/654 |  | 0.185 ± 0.096 | -0.702 ± 0.112 | -0.140 ± 0.093 | 0.734 ± 0.218 |
| pY766 |  |  | -0.887 ± 0.144 | -0.325 ± 0.132 | 0.549 ± 0.231 |
| pPLCγ |  |  |  | 0.562 ± 0.155 | 1.435 ± 0.241 |
| pFRS2 |  |  |  |  | 0.873 ± 0.229 |
| Downregulation |  |  |  |  |  |
| FGF8 | | | | | |
|  | pY653/654 | pY766 | pPLCγ | pFRS2 | Downregulation |
| pY653/654 |  | 0.116 ± 0.129 | -0.946 ± 0.135 | -0.753 ± 0.128 | 0.372 ± 0.150 |
| pY766 |  |  | -1.062 ± 0.118 | -0.870 ± 0.110 | 0.256 ± 0.136 |
| pPLCγ |  |  |  | 0.193 ± 0.126 | 1.318 ± 0.148 |
| pFRS2 |  |  |  |  | 1.125 ± 0.136 |
| Downregulation |  |  |  |  |  |
| FGF9 | | | | | |
|  | pY653/654 | pY766 | pPLCγ | pFRS2 | Downregulation |
| pY653/654 |  | 0.065 ± 0.152 | -0.956 ± 0.102 | 0.026 ± 0.079 | 0.734 ± 0.132 |
| pY766 |  |  | -1.020 ± 0.209 | -0.038 ± 0.202 | 0.670 ± 0.220 |
| pPLCγ |  |  |  | 0.982 ± 0.101 | 1.690 ± 0.145 |
| pFRS2 |  |  |  |  | 0.708 ± 0.123 |
| Downregulation |  |  |  |  |  |
