# Supplementary material for: Ligand bias underlies differential signaling of multiple FGFs via FGFR1
Source: eLife. 2024 Apr 3;12:RP88144. doi: 10.7554/eLife.88144 (PMC10990489; doi:10.7554/eLife.88144)
Supplement: Supplementary file 4. [file elife-88144-supp4.docx]

Supplementary table 4: Comparison of β’_4_, β’_8_, and β’_9_ values from Supplementary File 3. Shown are p values calculated by ANOVA to account for multiple comparisons. P values < 0.5 are highlighted in gray.

| β' Significance Values | | | |
| --- | --- | --- | --- |
|  | 4v8 | 4v9 | 8v9 |
| pY653/654 vs pY766 | >0.05 | >0.05 | >0.05 |
| pY653/654 vs pPLCγ | >0.05 | >0.05 | >0.05 |
| pY653/654 vs pFRS2 | 0.001 | >0.05 | 0.0002 |
| pY766 vs pPLCγ | >0.05 | >0.05 | >0.05 |
| pY766 vs pFRS2 | 0.025 | >0.05 | 0.0015 |
| pPLCγ vs pFRS2 | >0.05 | >0.05 | 0.0014 |
| pY653/654 vs DownRegulation | >0.05 | >0.05 | >0.05 |
| pY766 vs DownRegulation | >0.05 | >0.05 | >0.05 |
| pPLCγ vs DownRegulation | >0.05 | >0.05 | >0.05 |
| pFRS2 vs DownRegulation | >0.05 | >0.05 | >0.05 |
| Collagen Loss vs Growth Arrest | 0.0002 | 0.015 | 0.0007 |
