# Supplementary material for: Ligand bias underlies differential signaling of multiple FGFs via FGFR1
Source: eLife. 2024 Apr 3;12:RP88144. doi: 10.7554/eLife.88144 (PMC10990489; doi:10.7554/eLife.88144)
Supplement: Supplementary file 5. [file elife-88144-supp5.docx]

Supplementary table 5: Summary of results shown in Figures 5A-C.

|  | Endocytosis fluorescence | Viability slope | Apoptosis slope |
| --- | --- | --- | --- |
| FGF8 | 0.75 ±0.04 | -0.0001 ± 0.0001 | 0.0067 ± 0.0008 |
| FGF9 | 0.94 ±0.05 | -0.0017 ± 0.0003 | 0.0032 ± 0.0011 |
| No ligand | 1.00 ±0.04 |  |  |
